# Co-immobilization of amine dehydrogenase and glucose dehydrogenase for the biosynthesis of (*S*)-2-aminobutan-1-ol in continuous flow

**DOI:** 10.1186/s40643-024-00786-0

**Published:** 2024-07-18

**Authors:** Pengcheng Xie, Jin Lan, Jingshuan Zhou, Zhun Hu, Jiandong Cui, Ge Qu, Bo Yuan, Zhoutong Sun

**Affiliations:** 1 College of Biotechnology, Tianjin University of Science and Technology, Tianjin, 300457 China; 2 Tianjin Institute of Industrial Biotechnology, Chinese Academy of Sciences, Tianjin, 300308 China; 3 Key Laboratory of Engineering Biology for Low-Carbon Manufacturing, Chinese Academy of Sciences, Tianjin Airport Economic Area, 32 West 7th Avenue, Tianjin, 300308 China; 4 Institute of Industrial Catalysis, School of Chemical Engineering and Technology, Xi’an Jiaotong University, Xi’an Shaanxi, 710049 China

**Keywords:** Continuous flow, Asymmetric reductive amination, Amine dehydrogenases, Co-immobilization, Packed bed reactor

## Abstract

Reductive amination by amine dehydrogenases is a green and sustainable process that produces only water as the by-product. In this study, a continuous flow process was designed utilizing a packed bed reactor filled with co-immobilized amine dehydrogenase wh84 and glucose dehydrogenase for the highly efficient biocatalytic synthesis of chiral amino alcohols. The immobilized amine dehydrogenase wh84 exhibited better thermo-, pH and solvent stability with high activity recovery. (*S*)-2-aminobutan-1-ol was produced in up to 99% conversion and 99% ee in the continuous flow processes, and the space-time yields were up to 124.5 g L^-1^ d^-1^. The continuous reactions were also extended to 48 h affording up to 91.8% average conversions. This study showcased the important potential to sustainable production of chiral amino alcohols in continuous flow processes.

**Supplementary Information:**

The online version contains supplementary material available at 10.1186/s40643-024-00786-0.

## Introduction

Biocatalysis significantly contributes to a number of key research areas focusing on integrating green chemistry into the pharmaceutical industry. (Sheldon and Brady 2019) One of the challenges for applications of biocatalysis in the synthesis of key pharmaceutical building blocks is limited stability and recyclability of the enzymes, which can be alleviated by continuous flow processes. The utilization of immobilized enzymes and packed bed reactors (PBRs) in continuous flow processes represents major opportunities in biocatalysis field. (Basso and Serban 2019; Britton et al. 2018; Thompson et al. 2019b) Growing demands have been explored in pharmaceutical industry to replace the batch reactors with continuous flow processes. (Coloma et al. 2021; Cosgrove and Mattey 2022; Otvos and Kappe 2021; Plutschack et al. 2017; Sokač Cvetnić et al. 2023; Tamborini et al. 2018; Wan et al. 2022) Key benefits have been proposed including lower production cycle time, less waste, and reduced operating costs. (Brufani et al. 2023; De Santis et al. 2020) Particularly, in biocatalytic applications, continuous flow enables the reuse of immobilized enzymes in more cycles, in-situ removal of the products to reduce the product inhibition, (Meyer et al. 2022) and enhanced cofactor recyclability (Reus et al. 2024). Lipases (hydrolases, esterases), alcohol dehydrogenases, oxidases (glucose oxidases, galactose oxidases), (Cao et al. 2017; Chapman et al. 2018) ω-transaminases (TAs), (Andrade et al. 2014; Bohmer et al. 2019; Mattey et al. 2021) sugar derivatizing enzymes, HRPs (horse radish peroxidases), halohydrin dehalogenase, (Zhang et al. 2018) amidases, (Lin et al. 2019) and whole cell biocatalysts (Jin et al. 2019; Chen et al. 2022) have all been immobilized on various carriers and subjected to continuous flow processes.

Asymmetric reductive amination (ARA) is one of the prominent methodologies to produce chiral amines from the corresponding ketones or aldehydes. In particular, chiral vicinal amino alcohols are present in a variety of pharmaceuticals or natural products (Chen et al. 2019). Using free ammonia as the amine donor, amine dehydrogenases (AmDHs) are capable of catalyzing ARA of hydroxyl ketones, affording amino alcohols in high yields and ee. Ever growing research interests have focused on the identification and engineering of AmDHs to expand the substrate scope, improve the stability and activity of the enzymes. Since the first conversion of AmDHs from LeuDH by Bommarius et al. in 2012 (Abrahamson et al. 2012), a variety of AmDHs were converted from leucine dehydrogenases (LeuDHs) (Chen et al. 2018, 2019), phenylalanine dehydrogenases (PheDHs) (Jiang and Wang 2020; Li et al. 2022; Liu et al. 2020; Pushpanath et al. 2017; Ye et al. 2015), L-lysine dehydrogenases from *Geobacillus stearothermophilus* (LysEDHs) (Tseliou et al. 2019, 2021), or identified as native AmDHs (Bennett et al. 2022; Mayol et al. 2016, 2019). These investigations have inspired further applications of AmDH for the production of chiral amines as building blocks for pharmaceutical industry. Previously, we have performed genome mining and identified five AmDHs (*Gs*AmDH from *Geobacillus stearothermophilus*, *Bs*AmDH from *Bacillus stearothermophilus*, *Ls*AmDH from *Lysinibacillus sphaericus* CBAM5, *Sp*AmDH from *Sporosarcina psychrophile*, and *Ti*AmDH from *Thermoactinomyces intermedius*) that were able to catalyze the ARA of prochiral α- and β-hydroxy ketones. (Tong et al. 2021; Wang et al. 2020). Subsequently, protein engineering was conducted and high stereoselectivities and activities were obtained (Ming et al. 2022) towards the hydroxyl ketones to produce amino alcohols. These highly active mutants were subjected to ARA reactions under relatively large scale and high substrate concentrations, thus demonstrating the utility of the mutants for potential applications. Therefore, it is in urgent need to design and optimize immobilization techniques to enable continuous use of the enzymes in industrially viable packed bed reactors (PBR). However, the dependence on co-factors and recycling systems implies complexity for the applications of AmDHs. (Hu et al. 2022) The examples of using continuous enzymatic recycling NAD(P)H are scarce. (Croci et al. 2022; Romero-Fernandez and Paradisi 2021; Zhang et al. 2023; Zor et al. 2017) In particular, the specific activities of the immobilized enzymes (especially when immobilizing with crude enzymes or cell-free extracts) are often lower due to diffusion problems, variations in structures, or insufficient enzyme loading, etc. (Zhang et al. 2023) Though there have been reports for the immobilization strategies involving AmDHs, (Caparco et al. 2020; Liu et al. 2017; Ren et al. 2017) to date only a couple of studies have investigated the continuous flow processes with AmDHs, which afforded only moderate conversions. (Franklin et al. 2021; Thompson et al. 2019a)

**Fig. 1:**
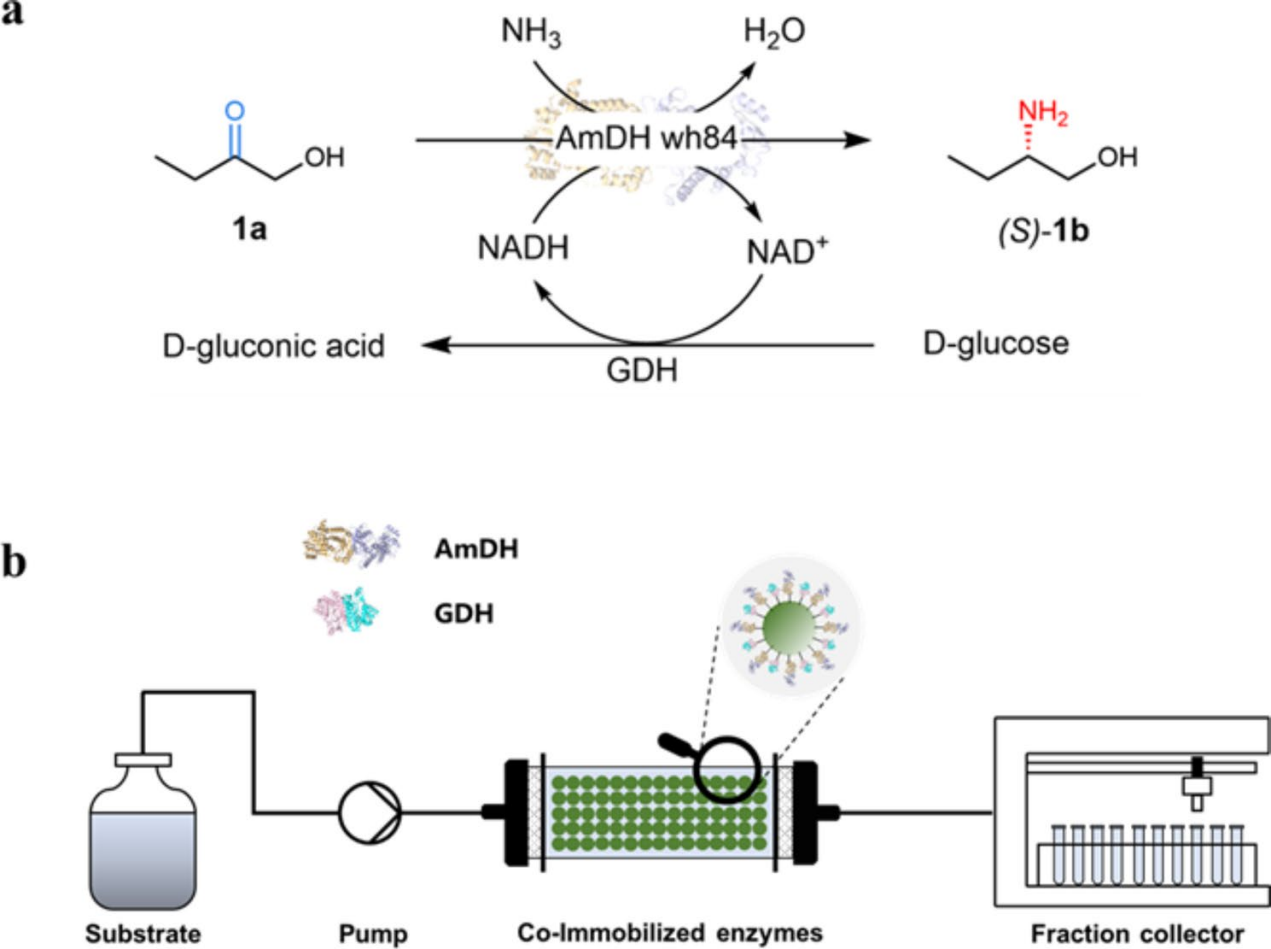
(**a**), Reductive amination of 1-hydroxybutan-2-one (**1a**); (**b**), Representative diagram for the continuous flow process

Herein, we envisioned a continuous flow process using PBRs with co-immobilized variant of AmDH wh84 and GDH for the co-factor recycling of the reductive amination of 1-hydroxybutan-2-one (**1a**) to produce the optically active (*S*)-2-aminobutan-1-ol ((*S*)-1b) (Fig. 1 and Scheme S1). The co-immobilization of AmDH wh84 and GDH on polymer-based porous bead carriers was characterized and optimized, and subsequently the advantages of the continuous flow processes were demonstrated with comparison of batch reactions. This study showcases the potentials of using co-immobilized AmDHs and GDH in the industrial processes with continuous flow and offers paradigm for the future design of large-scale bioprocesses.

## Experimental section

### Materials

An engineered strain, AmDH wh84, was constructed from our previous work and used in the current study. Resin carriers were purchased from Xi’an Lan Xiao Technology Co., Ltd., (Xi’an, China) and Tianjin Nankai He Cheng Technology Co., Ltd. (Tianjin, China). The magnetic nanoparticles were purchased from Yeasen Biotechnology Co. Ltd. (Shanghai, China). 1-hydroxy-2-butanone, 2-amino-1-butanol and other chemicals were purchased from Shanghai Macklin Biochemical Technology Co., Ltd and Bide Pharmatech Ltd.

### Preparation of crude extracts of AmDH and GDH

The genes encoding AmDH or GDH were cloned into the commercially available pET-24a vector containing the NdeI and XhoI restriction sites, and were constructed with an N-terminal 6xHis tag. The transformed *E. coli* BL21(DE3) cells were stored in 50% glycerol at -80 °C. To initiate protein expression, cells were thawed, inoculated into 5 mL LB medium containing 50 µg mL^-1^ kanamycin, and incubated at 37 °C with shaking at 220 rpm for 8–12 h. A 1 mL aliquot of the seed culture was then transferred to 500 mL of TB medium containing 50 µg mL^-1^ kanamycin and grown at 37 °C with shaking at 220 rpm until an OD_600_ of 0.6–0.8 was reached. Protein expression was induced by adding 0.1 mM isopropyl β-D-1-thiogalactopyranoside (IPTG) and incubating the cells at 20 °C with shaking at 220 rpm for 15 h. The cells were collected by centrifugation (4 °C, 4000 rpm, 10 min) and resuspended in PBS buffer (50 mM, pH 7.4), followed by centrifugation to remove the supernatant. The cells were then resuspended in the same buffer at a concentration of 0.1 g ml^-1^ (cell wet weight). Subsequently, the cells were disrupted using a high-pressure cell homogenizer and centrifuged (4 °C, 12,000 rpm, 40 min) to remove the precipitates, resulting in crude protein extracts of AmDH and GDH.

### Immobilization of enzymes on amino resins

First, 6.0 g amino resin was washed three times with 24 mL of PBS buffer (50 mM, pH 7.4), and then the resin carriers were filtered and dried under reduced pressure. Next, the resulting amino resins were mixed with 24 mL of 2% glutaraldehyde solution at 20 °C for 1 h and subsequently washed by PBS buffer. Finally, the resins were mixed with 24 mL crude protein extract and stirred at 200–250 rpm, 20 °C for 18 h, followed by 1 h of settling. The supernatant was collected to determine the protein loadings and immobilization yields. The immobilized enzymes were washed with PBS buffer (50 mM, pH 7.4) two to three times, filtered and stored for further experiments.

### Immobilization procedures of enzymes on epoxy resins

Firstly, 6.0 g epoxy resin was washed three times with 24 mL PBS buffer (50 mM, pH 7.4), and then the resin carriers were filtered and dried under reduced pressure. Next, the resulting epoxy resins were mixed with 24 mL crude protein extract and stirred at 200–250 rpm, 20 °C for 18 h, followed by 1 h of settling. The supernatant was collected to determine the protein loadings and immobilization yields. Finally, the immobilized enzymes were washed with PBS buffer (50 mM, pH 7.4) two to three times, filtered and stored for further experiments.

### Enzyme assays

Conditions for enzyme activity analysis of AmDH: 200 mg co-immobilized AmDH and GDH or 200 µL AmDH (crude extract) and 50 µL GDH (crude extract), 1 mM NAD^+^, 100 mM glucose, 50 mM Tris-HCl (pH 9.5) buffer (including 1 M NH_4_Cl), 10 mM **1a**, 30 °C, 30 min, total volume: 1 mL. The reaction was then terminated using acetonitrile. The product (*S*)-**1b** was quantified by HPLC after derivatization with Marfey’s reagent (N-alpha-(2,4-dinitro-5-fluorophenyl)-L-alaninamide). Detection conditions (Li et al. 2017): Zorbax SB-C18 column (4.6*150 mm, 5 μm), 340 nm wavelength, with 25 ℃ column temperature, at 1 mL min^-1^ flow rate; mobile phase A: ultrapure water, mobile phase B: methanol. Elution program: 60% A/40% B, hold at 40% B for 6 min, increase B to 60% within 9 min, hold for 3 min, then decrease B to 40% within 2 min, hold for 5 min. One unit (U) of activity was defined as the amount of enzyme required to produce 1 µmol of (*S*)-**1b** per minute under the standard assay conditions.

The activity recovery (AR) is defined as the ratio of the immobilized enzymatic activity over the total free enzymatic activity added to the reaction mixture for the immobilization. Immobilized activity (IA) defined as the activity of AmDH per gram of co-immobilized enzyme. The protein loading (PL) is defined as the mass of enzyme immobilized on one gram of resins. The immobilization yield (IY) is defined as the ratio between the mass of immobilized enzyme over the total free enzyme added to the reaction mixture for the immobilization. The activity of GDH (U_GDH_) is determined by measuring the change in absorbance values of NADH at 340 nm by UV spectrophotometry. Conditions: 10 µL GDH (crude extracts), 10 mM NAD^+^, 100 mM glucose and 50 mM Tris-HCl (including 1 M NH_4_Cl) buffer (pH 9.5), total volume: 200 µL, 30 ℃. One unit (U) of GDH activity is defined as the amount of enzyme required to produce 1 µmol of NADH per minute under the standard assay conditions. The definitions can also be interpreted by the following equations:

1 $${\rm{AR(\% ) = }}{{{A_i}} \over {{A_0}}}{\rm{ \times 100\% }}$$

2 $${\rm{IA\,(U/}}{{\rm{g}}_{{\rm{resin}}}}{\rm{) = }}{{Ai} \over {{m_{re\sin }}}}$$

3 $${\rm{PL}}\,{\rm{(mg/g) = }}{{{m_0} - {m_s}} \over {{m_{re\sin }}}}$$

4 $${\rm{IY}}\,{\rm{(\% ) = }}{{{m_0} - {m_s}} \over {{m_0}}} \times {\rm{100\% }}$$

5 $${{\rm{U}}_{{\rm{GDH}}}} = {{EW{\rm{ \times }}V{\rm{ \times 1}}{{\rm{0}}^{\rm{3}}}} \over {M{\rm{ \times }}L}}$$

Where *A*_*i*_ is the immobilized AmDH activity, U; *A*_*0*_ is the total free AmDH activity added to the reaction mixture for the immobilization, U; *m*_*resin*_ is the mass of resin, g; *m*_*0*_ is the mass of the total free enzyme, mg; *m*_*s*_ is the mass of enzyme in the supernatant after immobilization, mg; *EW* is the change in absorbance at 340 nm within 1 min; *V* is the volume of the reaction solution, mL; M is the molar absorption coefficient of the NADH, L mol^-1^ cm^-1^; L is the optical distance, cm.

### Enzyme stability

To determine the stability of immobilized enzymes, they were incubated for 2 h under various pH values (3.0–13.0), temperatures (℃), and organic solvents such as methanol, acetonitrile, n-hexane, N, N-dimethylformamide (DMF), dimethyl sulfoxide (DMSO), methyl *tert-*butyl ether (MTBE), ethyl acetate to evaluate the pH stability, thermo-stability, and organic solvent tolerance of immobilized and free enzymes. The enzyme activity measured at pH 9.5 and 30 ℃ is defined as 100% to determine the relative activity.

### Batch reactions of immobilized enzymes

200 mg immobilized enzymes were added to 1 ml Tris-HCl (including 1 M NH_4_Cl) buffer (50 mM, pH 9.5) containing 10 mM **1a**, 1 mM NAD^+^ and 100 mM glucose. Buffer pH was adjusted by KOH or HCl. The mixture was then incubated with agitation at 30 ℃ and 220 rpm for 0.5 to 2 h. The generated products were quantified by HPLC.

### AmDH/GDH binding ratio optimization

Six different ratios of AmDH and GDH enzymes were immobilized using the LXTE-706 carrier. For each ratio, 6.0 g of LXTE-706 carrier were mixed with 24 mL of crude protein extracts of AmDH and GDH for the immobilization reaction using the epoxy resin immobilized enzyme method described above. Protein loading (PL) and immobilization yield (IY) were determined by measuring initial protein concentrations and the concentrations in the supernatant after completion of the immobilization reaction. To test the immobilized enzymes, 200 mg of each were added to 1 mL Tris-HCl (including 1 M NH_4_Cl) buffer (50 mM, pH 9.5) containing 10 mM **1a**, 1 mM NAD^+^, and 100 mM glucose. The mixture was incubated with agitation at 30 ℃ and 220 rpm for 0.5 h, and conversions were determined by HPLC.

### Continuous reactions with a fixed-bed reactor

Immobilized enzyme was filled in a fix-bed reactor (Column Y2, 7.85 mL volume). A peristaltic pump was used to pump Tris-HCl (including 1 M NH_4_Cl) buffer (50 mM, pH 9.5) containing 10 mM **1a**, 1 mM NAD^+^ and 100 mM glucose into the packed bed bioreactor. Buffer pH was adjusted by KOH or HCl. 2 column volumes (CV) of the solution mixture were allowed to flow through the column before the reactions reached steady state and conversions reached plateau. Subsequently, the samples were collected in the fraction collector and analyzed using HPLC.

## Results and discussion

### Tailored co-immobilization system for AmDH and GDH

AmDHs are NADH dependent enzymes, which require co-factor recycling systems (e.g. GDH and glucose) to regenerate the co-factors. Therefore, to reduce the overall cost for enzymes and enable the reuse of both AmDH and GDH on PBRs, it is only economically viable if both enzymes are immobilized. Previously, we successfully identified, characterized and engineered an amine dehydrogenase from *Sporosarcina psychrophila* (*Sp*AmDH) derived from leucine dehydrogenase. (Tong et al. 2021) Upon three rounds of CAST/ISM-guided mutagenesis, the mutant wh84 was constructed with 3.2-fold improvement in TTN to the WT enzyme. In this work, we cultured and expressed the variant AmDH wh84 and the crude extract was used for the immobilization directly without enzyme purification. Additionally, glucose dehydrogenase (GDH) from *Bacillus subtilis* was also expressed and immobilized as a crude extract.

A number of immobilization strategies were investigated on the purpose of selecting the best-performing carriers for co-immobilization. The commercially available ion-exchange, amino and epoxy resins as well as magnetic nanoparticles with carboxyl groups modified surfaces were utilized. We characterized the properties of co-immobilized enzymes on various carriers in terms of immobilization activity (IA), activity recovery (AR), Protein loading (PL), Immobilization yield (IY) of AmDH wh84, pore sizes and particle sizes (Table 1). Among the carriers, the epoxy resin LXTE-706, ion-exchange resin ESQ-3and LXTE-902, and the amino resin LXTE-700s exhibited highest immobilization activity (IA up to 1.35 U g^-1^) and activity recovery (AR up to 55.30%). The magnetic nanoparticles offer advantages such as ease for separation and strong covalent bonding with surface modifications, however, the activity was significantly lower than that of the resins and not pursued further (Table S1). In the end, the resins LXTE-700s, LXTE-706, ESQ-3, LXTE-902 were selected as the carriers for the subsequent process development and optimizations.

**Table 1:** The comparisons of co-immobilized AmDHs and GDH on various carriers

Carrier type	Model	Specific activity (IA)^a^ [U g^− 1^]	Activity recovery (AR)^b^ [%]	Protein loading (PL)^c^ [mg g^− 1^]	Immobilization yield (IY)^d^ [%]	Particle sizes [µm]	Pore diameters [Å]
Ion exchange resin	LXTE-902	1.35 ± 0.03	55.3 ± 1.23	249.6	80.0	150–350	400–600
	ESQ-3	1.33 ± 0.025	54.3 ± 1.02	163.2	52.3	100–300	100–300
Amino resin	LXTE-700s	0.69 ± 0.05	28.3 ± 2.05	129.6	41.5	300–500	200–400
	LXTE-700	0.09 ± 0.01	3.7 ± 0.01	134.4	43.1	150–300	200–400
	LXTE-701	0.26 ± 0.01	10.7 ± 0.41	187.2	60.0	100–300	500–700
	LXTE-703	0.25 ± 0.01	10.2 ± 0.01	172.8	55.4	100–250	300–500
	LXTE-704	0.28 ± 0.01	11.5 ± 0.41	158.4	50.8	100–300	500–700
Epoxy resin	LXTE-706	1.33 ± 0.015	54.3 ± 0.61	159.2	51.0	100–300	100–300
	LXTE-603	0.01 ± 0.001	0.4 ± 0.01	62.6	20.1	100–300	280–320
	LXTE-604	0.05 ± 0.005	1.8 ± 0.20	62.6	36.9	100–300	270–320
	LXTE-609	0.02 ± 0.005	0.6 ± 0.20	115.2	18.5	100–300	260–320

a, IA was defined by Eq. (2); b, AR was defined by Eq. (1); c, PL is defined by Eq. (3) and IY is defined by Eq. (4)

The AmDHs (wh84) / GDH ratio is 5/1

The enzyme activities for GDH and AmDH are highly different, as a result, the optimum ratios of AmDHs to GDH were investigated using LXTE-706 on the purpose of lowering enzyme usage and balancing enzymatic activities for the co-factor recycling. The specific enzyme activity of the GDH crude extract were determined to be 5.50 U mL^-1^, which was significantly higher than that of AmDH (0.61 U mL^-1^). Indeed, with an AmDH/GDH ratio 5:1, the conversion reached a maximum of 80.5% (Table 2). Further increasing the ratio did not lead to higher conversions. Therefore, 5:1 ratio was employed in the following studies.

**Table 2:** Optimization of AmDH/GDH ratios

AmDH/GDH ratios*	Protein loading (PL)^a^ [mg g^-1^]	Immobilization yield (IY)^a^ [%]	Conv. [%]
6: 1	148.8	46.3	52.0 ± 1.7
5: 1	144.0	44.8	80.5 ± 4.0
2: 1	158.4	49.3	67.1 ± 0.2
1: 1	148.8	46.3	60.4 ± 0.4
1: 2	168.0	52.2	41.3 ± 0.6
1: 5	158.4	49.3	29.7 ± 2.5

* Co-immobilized on the carrier LXTE-706. a, PL is defined by Eq. (3) and IY is defined by Eq. (4)

There are two strategies for the immobilization of the enzymes: separate immobilization or co-immobilization. For the separate immobilization strategy, the enzymes were immobilized on carriers separately and the resins carrying each of the enzyme were mixed and added to the reactions. On the other hand, the crude enzymes of AmDH wh84 and GDH were co-immobilized on the carriers in one-step. The resulting immobilization activity (IA) of AmDH wh84 achieved for the co-immobilization strategy was 0.69 U g^-1^, which was much higher than that of the separate immobilization (0.43 U g^-1^, Fig. S4a). Additionally, the co-immobilization reduced the steps for immobilization and operational costs, therefore, co-immobilized AmDH wh84 and GDH were employed for further investigations.

The SDS-PAGE gel analysis was performed on the mixture of crude extract of AmDH wh84 and GDH and the supernatant after the immobilization process was performed for 3–18 h (Fig. S4b). There were significantly less enzymes compared to the crude enzyme mixture, which was in accordance with the immobilization yields (IY up to 52.2%). The crude enzymes were used for immobilization directly without further purification for the ease of operation and cost saving. The surface Lysine distribution was visualized (Fig. S3), which consists of 4.8% and 6.1% of total amino acids of AmDH and GDH (Table S2).

**Fig. 2:**
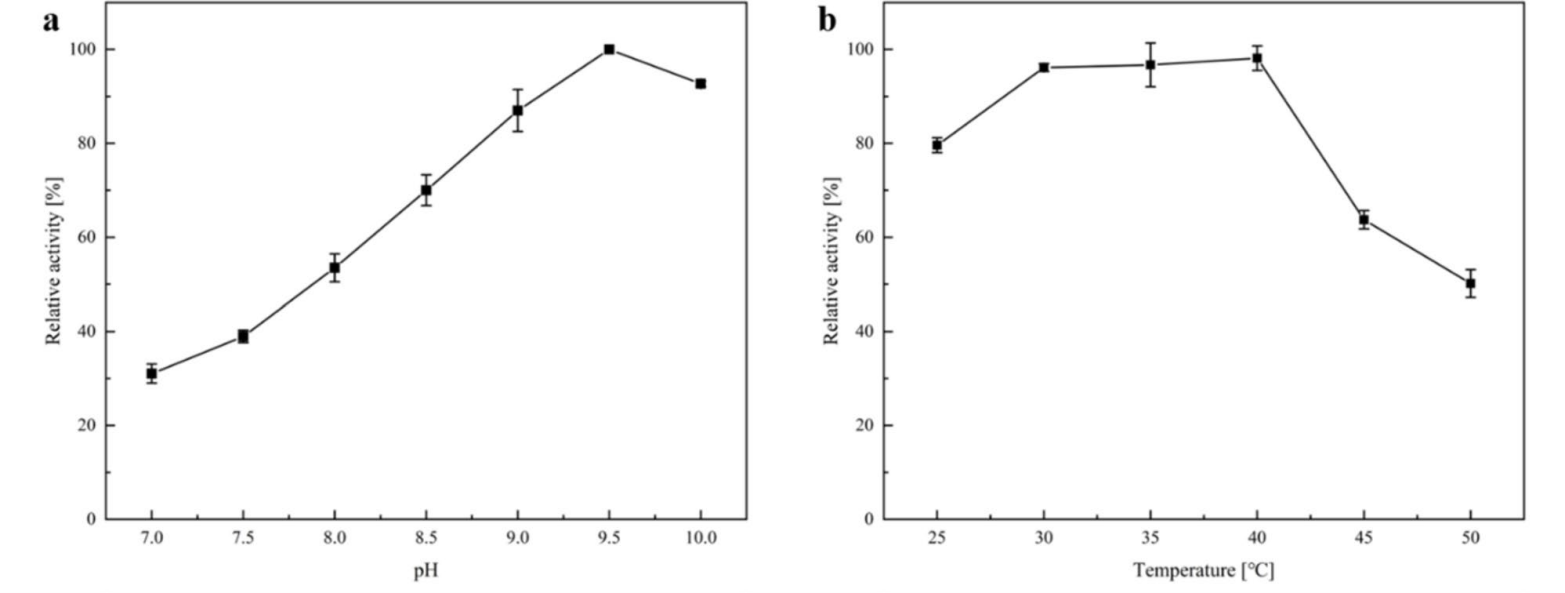
Optimization of the batch reactions with co-immobilized enzymes. (**a**), Optimization of pH for the batch reactions using co-immobilized AmDH wh84 and GDH. Buffer pH was adjusted by KOH or HCl. Reaction conditions: NAD^+^ (1 mM), glucose (100 mM), Tris-HCl (50 mM, pH 7.0–10.0) buffer (including 1 M NH_4_Cl), immobilized enzymes (200 mg), **1a** (10 mM), 30 °C, total volume: 1 mL, carrier: LXTE-706; (**b**), Optimization of reaction temperatures for the batch reactions using co-immobilized AmDH wh84 and GDH. Reaction conditions: NAD^+^ (1 mM), glucose (100 mM), Tris-HCl (50 mM, pH 9.5) buffer (including 1 M NH_4_Cl), immobilized enzymes (200 mg), **1a** (10 mM), 25–50 °C, total volume: 1 mL, carrier: LXTE-706

The co-immobilized enzymes were employed in the batch reactions to convert **1a** to (*S*)-**1b**. The optimum pH was determined to be 9.5 after performing the reactions in various pH (Fig. 2a). Various reaction temperatures were also attempted to determine the optimum conditions, and 30–40 °C were shown to be the best range (Fig. 2b) It is worth noting that the optimal reaction conditions for free AmDH wh84 are pH 8.5 and 30 °C. In the end, the optimum pH 9.5 and 30 °C temperature was employed in further investigations.

### Batch reactions with co-immobilized AmDH and GDH

**Fig. 3:**
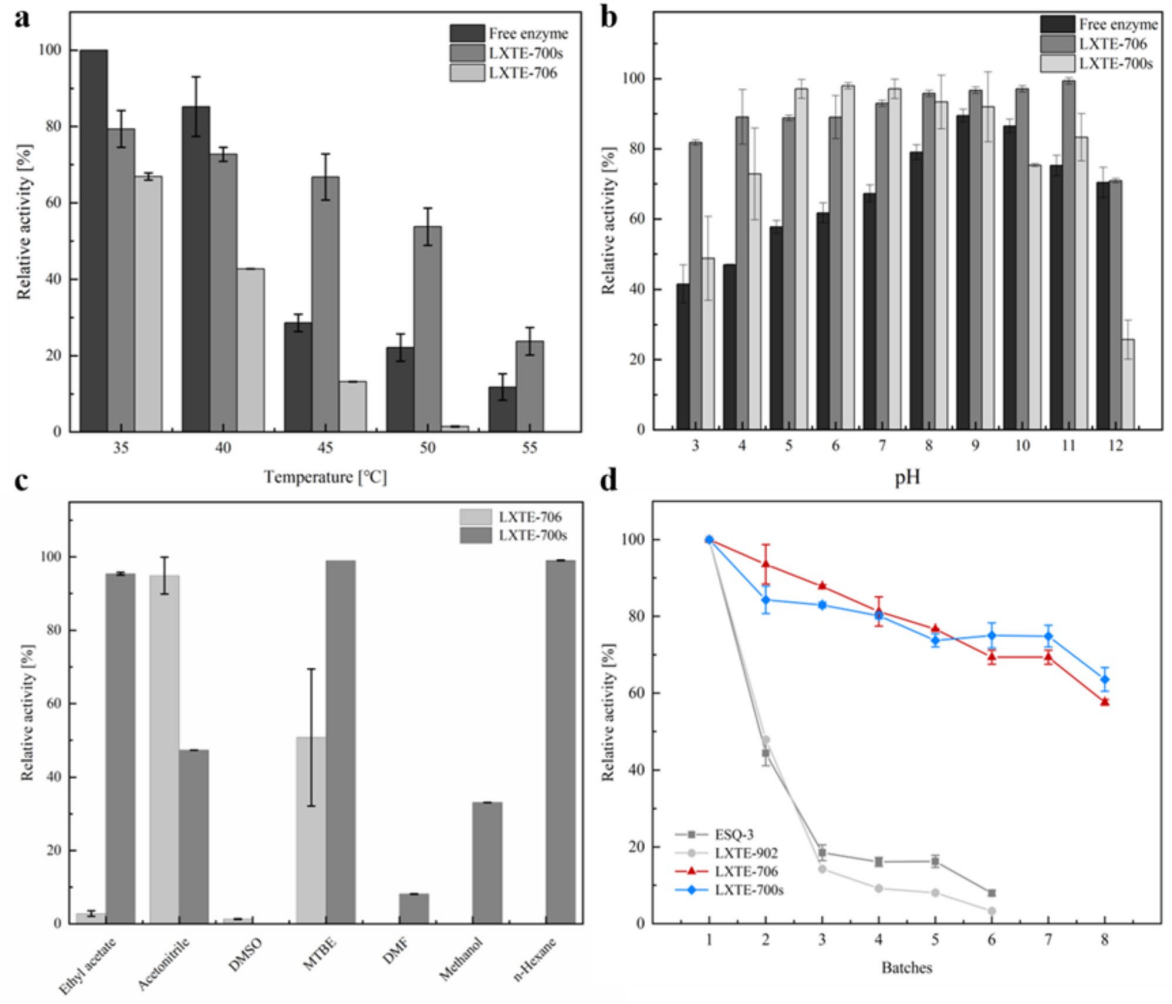
Characterizations of the co-immobilized enzymes. (**a**), Thermo-stability of the free and co-immobilized enzymes; (**b**), pH stability of the free and co-immobilized enzymes; (**c**), Solvent tolerance of co-immobilized enzymes; (**d**), Reusability of the enzymes co-immobilized on various carriers. Reaction conditions: NAD^+^ (1 mM), glucose (100 mM), Tris-HCl (including 1 M NH_4_Cl) buffer (50 mM, pH 9.0 for LXTE-700s, or pH 9.5 for LXTE-706), immobilized enzymes (200 mg), **1a** (10 mM), 30 °C, total volume 1 mL, incubation time: 2 h

Thermostability and pH stability of the co-immobilized enzymes and free enzymes were investigated by incubation at various temperatures (30–55 °C) (Fig. 3a) and pH (3.0–12.0) (Fig. 3b) for two hours and the residual activities were tested. For temperatures at 30–40 °C, the relative activity for the free enzymes were higher than that of the immobilized enzymes, however, at an elevated temperature of 45–55 °C, the free enzymes lost a majority of the activity, whereas the co-immobilized enzymes were able to retain 30-40% activity (LXTE-706) and 40-70% activity (LXTE-700s), respectively. Furthermore, the immobilized enzymes displayed considerably higher pH stability than free enzymes at extreme pH values such as pH 3.0–8.0 and 11.0–13.0, indicating higher stability of the immobilized enzymes in acidic or basic conditions. At pH 9.0–11.0, the immobilized enzymes on LXTE-706 were more active than LXTE-700s and the free enzymes, showing the distinct advantages of the immobilization processes in improving the pH stability of the enzymes.

Organic solvents are frequently used in biocatalytic processes to aid the solvation of substrates and products; however, enzymes were shown to be unstable in presence of solvents possibly due to unfolding, denaturation or aggregation. Immobilization technologies may offer elegant solutions to solve the solvent intolerance problems. Therefore, even though the current strategy does not involve the use of organic solvents, for potential future applications with substrate with poor water solubility, we investigated the stability of the co-immobilized enzymes on carriers including LXTE-706 with the highest immobilization AmDH activity (1.33 U g^-1^), and LXTE-700s with higher thermo- and pH stability. Upon incubation in various solvents for two hours with different log *P* values, their residual activities were compared (Fig. 3c). The co-immobilized enzymes on LXTE-700s retained > 90% activity in solvents including ethyl acetate, n-hexane and MTBE. On the other hand, LXTE-706 as the carrier exhibited lower activities in most of the solvents, except that in acetonitrile, where the activity was 48% higher than that of the LXTE-700s.

The co-immobilized enzymes in batch reactions can be recycled and reused for various batches. Filtration followed by buffer wash were performed on the immobilized enzymes prior to each batch reactions. Highest activities were obtained for enzymes immobilized on LXTE-700s and LXTE-706. 63.6% and 57.5% activity were retained after 8 batches of reuse, respectively (Fig. 3d). In comparison, ESQ-3 as the carrier offered one of the highest activity recoveries (AR up to 54.3%), but the activity declined rapidly and completely lost after 6 batches of reuse. Poor reusability was also observed for enzymes immobilized on LXTE-902. The enzymes were immobilized with stable covalent bindings on LXTE-700s and LXTE-706, which are possibly the reason why the recyclability exceeds that of the ion exchange resins LXTE-902 and ESQ-3. In addition, the LXTE-700s carrier is functionalized with amino groups on the surface, and LXTE-706 is functionalized with both epoxy and amino groups. Since epoxy groups are prone to ring-opening and losing functionality upon extended exposure to air, we speculate that immobilized enzymes on LXTE-700s are more stable due to more stable linkage established with amino surface groups.

### Continuous flow biocatalysis with co-immobilized enzymes

**Fig. 4:**
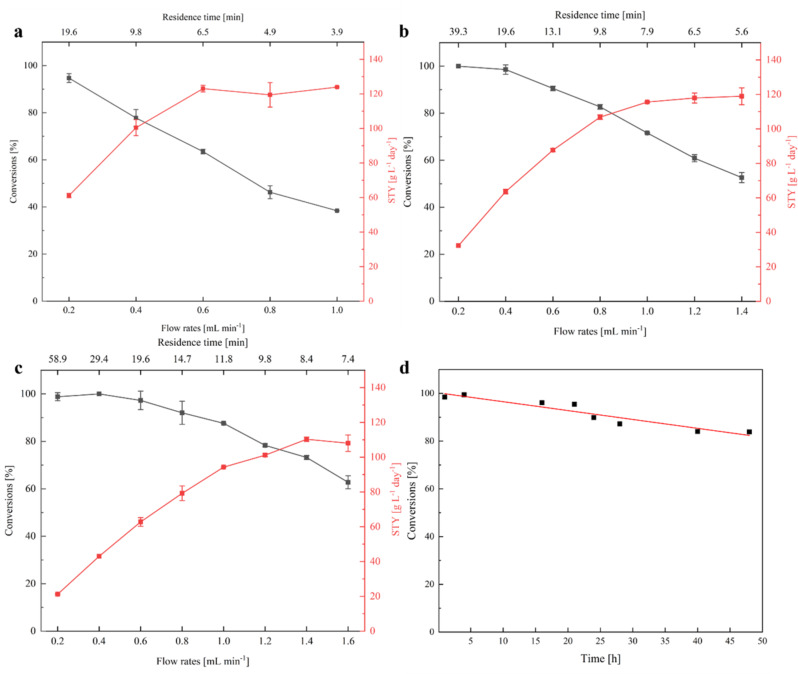
Comparisons of the continuous flow processes under various flow rates with (**a**), Column Y1; (**b**), Column Y2; (**c**), Column Y3; (**d**), Conversions for the continuous flow reactions in 48 h (Column Y2, 0.2 ml min^-1^ flow rate). Reaction conditions: NAD^+^ (1 mM), glucose (100 mM), Tris-HCl **(**including 1 M NH_4_Cl) buffer (50 mM, pH 9.5), **1a** (10 mM), 30 °C, carrier: LXTE-706

Enzymatic ARA of **1a** was carried out in PBRs filled with co-immobilized AmDHs wh84 and GDH, and the carrier LXTE-706 was selected considering both the immobilization activity and stability. Subsequently, we performed the continuous flow studies under various flow rates (0.2 mL min^-1^ to 1.6 mL min^-1^). A fraction collector and subsequent HPLC analysis enabled the monitoring of the results (Fig. S1-S2, S5-S8). Owing to excellent enantioselectivity of AmDHs, ee of (*S*)-**1b** maintained > 99% throughout the studies. Three columns with the same cross-sectional area and different lengths (Y1: 10*50 mm, Y2: 10*100 mm, Y3: 10*150 mm) were utilized to compare the space-time-yields (STY) and conversions under various flow rates. The residence times at different flow rates are labeled in Fig. 4 and listed in Table S3. The STY for whole-cell biotransformation in batch preparative scale (200 mM, 91% conversions in 24 h, 0.1 g mL^-1^ wet cell) was also compared with the continuous flow reactions. (Tong et al. 2021) Subsequently, we performed the continuous flow studies under various flow rates (0.2 mL min^-1^ to 1.6 mL min^-1^). With samples taken after the reaction reached steady state at various flow rates, the maximum STY for the continuous reactions reached 124.5, 122.4, and 111.4 g L^-1^ d^-1^ for columns Y1, Y2 and Y3, respectively (Fig. 4a-c). As a result, at 99% conversion, the maximum STY for the continuous flow reaction (64.6 g L^-1^ d^-1^) is up to 4-fold to that of the whole-cell batch reaction (16.2 g L^-1^ d^-1^) and the immobilized enzyme in batch reaction (16.0 g L^-1^ d^-1^). Up to 99% conversions and 99% ee were achieved with Column Y2 and Y3 at low flow rates (0.2–0.4 mL min^-1^). The STY exhibits a gradual increase at relatively low flow rates, and eventually reaches plateau at higher flow rates for all three columns. In addition, at approximately 6.5 min residence time, the STY for Y1 and Y2 are close to 120.0 g L^-1^ d^-1^. The results indicate that the reactions were influenced by external diffusion at low flow rates, and this effect were minimized at higher flow rates. The STYs achieved in this study is relatively high at this scale comparing to previous studies (Table S4), which were mostly performed at small scales.

The continuous flow reaction was extended to 48 h to achieve an upscaled production of (*S*)-**1b** and examine the stability of the co-immobilized enzymes (Fig. 4d). The column Y2 and 0.2 ml min^-1^ flow rate were selected, and the conversions maintained in the range of 83.8–99.0% throughout 48 h. The average conversions reached 91.8%, producing 470.5 mg of the product (*S*)-**1b**. The residual activity for the immobilized AmDH was determined to be 1.01 U g^-1^, which is 75.9% of the immobilization activity of the fresh enzyme (1.33 U g^-1^). The STY of the continuous system exceeds that of the whole-cell biotransformation and batch reactions, and the immobilized enzymes were not directly agitated to avoid the physical destruction. In particular, the recyclability is enhanced as shown from the results of the 48 h continuous production. Therefore, from economic perspective, the continuous flow system affords reduced cost and improved recyclability. The current system is also easy to scale-up, as demonstrated by a similar final STY achieved with varied lengths and filling capacity of columns. We envisioned that the current system may offer a useful platform for preparative scales for sustainable production of chiral amino alcohols.

## Conclusions

In this work, the enzymes AmDH wh84 and GDH were co-immobilized on various carriers and characterized by thermo-, pH, solvent stability and reusability. Continuous flow biocatalysis were investigated utilizing three different columns and the maximum STY was up to 124.5 g L^-1^ d^-1^. At 99.0% conversion, the maximum STY for the continuous flow reaction is up to 4-fold to that of the whole-cell batch reactions and batch reactions with immobilized enzymes. The average conversions for the 48 h continuous flow process reached 91.8%, producing 470.5 mg product in excellent enantioselectivity. The reactions proceed in aqueous buffers without the use of organic solvents and only produce water as the main by-product. Therefore, the current study represents an encouraging contribution to the sustainable industrialization for enzymatic production of chiral amino alcohols by continuous flow processes.

### Electronic supplementary material

Below is the link to the electronic supplementary material.

Supplementary Material 1

Supplementary Material 2

## Data Availability

All data generated or analyzed during this study are included in this published article (and its supplementary information files).

## References

[CR1] Abrahamson MJ, Vázquez-Figueroa E, Woodall NB, Moore JC, Bommarius AS (2012). Development of an amine dehydrogenase for synthesis of chiral amines. Angew Chem Int Ed.

[CR2] Andrade LH, Kroutil W, Jamison TF (2014). Continuous flow synthesis of chiral amines in organic solvents: immobilization of e. coli cells containing both omega-transaminase and PLP. Org Lett.

[CR3] Basso A, Serban S (2019) Industrial applications of immobilized enzymes—A review. Mol Catal 479. 10.1016/j.mcat.2019.110607

[CR4] Bennett M, Ducrot L, Vergne-Vaxelaire C, Grogan G (2022) Structure and mutation of the native amine dehydrogenase MATOUAmDH2. 10.1002/cbic.202200136. Chembiochem 23 10.1002/cbic.202200136 PMC9325545 35349204

[CR5] Bohmer W, Knaus T, Volkov A, Slot TK, Shiju NR, Cassimjee KE, Mutti FG (2019). Highly efficient production of chiral amines in batch and continuous flow by immobilized omega-transaminases on controlled porosity glass metal-ion affinity carrier. J Biotechnol.

[CR6] Britton J, Majumdar S, Weiss GA (2018). Continuous flow biocatalysis. Chem Soc Rev.

[CR7] Brufani G, Valentini F, Rossini G, Rosignoli L, Gu Y, Liu P, Vaccaro L (2023). Waste-minimized continuous flow copper-catalyzed azide-alkyne cycloaddition with low metal contamination. Green Synth Catal.

[CR8] Cao S-L, Xu H, Lai L-H, Gu W-M, Xu P, Xiong J, Yin H, Li X-H, Ma Y-Z, Zhou J, Zong M-H, Lou W-Y (2017). Magnetic ZIF-8/cellulose/Fe3O4 nanocomposite: preparation, characterization, and enzyme immobilization. Bioresour Bioprocess.

[CR9] Caparco AA, Bommarius BR, Bommarius AS, Champion JA (2020). Protein-inorganic calcium-phosphate supraparticles as a robust platform for enzyme co-immobilization. Biotechnol Bioeng.

[CR10] Chapman MR, Cosgrove SC, Turner NJ, Kapur N, Blacker AJ (2018). Highly productive oxidative biocatalysis in continuous flow by enhancing the aqueous equilibrium solubility of oxygen. Angew Chem-Int Edit.

[CR12] Chen F-F, Zheng G-W, Liu L, Li H, Chen Q, Li F-L, Li C-X, Xu J-H (2018). Reshaping the active pocket of amine dehydrogenases for asymmetric synthesis of bulky aliphatic amines. ACS Catal.

[CR11] Chen F-F, Cosgrove SC, Birmingham WR, Mangas-Sanchez J, Citoler J, Thompson MP, Zheng G-W, Xu J-H, Turner NJ (2019). Enantioselective synthesis of chiral vicinal amino alcohols using amine dehydrogenases. ACS Catal.

[CR13] Chen Q-S, Yuan X, Peng F, Lou W-Y (2022). Immobilization of engineered E. Coli cells for asymmetric reduction of methyl acetoacetate to methyl^®^3-hydroxybutyrate. Bioresour Bioprocess.

[CR14] Coloma J, Guiavarc’h Y, Hagedoorn P-L, Hanefeld U (2021). Immobilisation and flow chemistry: tools for implementing biocatalysis. Chem Commun.

[CR15] Cosgrove SC, Mattey AP (2022). Reaching New Biocatalytic reactivity using continuous flow reactors. Chemistry.

[CR16] Croci F, Vilim J, Adamopoulou T, Tseliou V, Schoenmakers PJ, Knaus T, Mutti FG (2022). Continuous flow biocatalytic reductive amination by co-entrapping dehydrogenases with agarose gel in a 3d-printed mould reactor. ChemBioChem.

[CR17] De Santis P, Meyer LE, Kara S (2020). The rise of continuous flow biocatalysis - fundamentals, very recent developments and future perspectives. REACT CHEM ENG.

[CR18] Franklin RD, Whitley JA, Caparco AA, Bommarius BR, Champion JA, Bommarius AS (2021). Continuous production of a chiral amine in a packed bed reactor with co-immobilized amine dehydrogenase and formate dehydrogenase. Chem Eng J.

[CR19] Hu Y-J, Chen J, Wang Y-Q, Zhu N, Fang Z, Xu J-H, Guo K (2022). Biocatalysts used for multi-step reactions in continuous flow. Chem Eng J.

[CR20] Jiang W, Wang Y (2020). Improving catalytic efficiency and changing substrate spectrum for asymmetric biocatalytic reductive amination. J Microbiol Biotechnol.

[CR21] Jin LQ, Yang B, Xu W, Chen XX, Jia DX, Liu ZQ, Zheng YG (2019). Immobilization of recombinant Escherichia coli whole cells harboring xylose reductase and glucose dehydrogenase for xylitol production from xylose mother liquor. Bioresour Technol.

[CR22] Li B, Zhang J, Yang B-B, Li L, Yang X-X (2017). Ring-locking strategy facilitating determination of absolute optical purity of 2-amino-1-butanol by reverse-phase high-performance liquid chromatography. RSC Adv.

[CR23] Li J, Mu X, Wu T, Xu Y (2022). High coenzyme affinity chimeric amine dehydrogenase based on domain engineering. Bioresour Bioprocess.

[CR24] Lin CP, Wu ZM, Tang XL, Hao CL, Zheng RC, Zheng YG (2019). Continuous production of aprepitant chiral intermediate by immobilized amidase in a packed bed bioreactor. Bioresour Technol.

[CR25] Liu J, Pang BQW, Adams JP, Snajdrova R, Li Z (2017). Coupled immobilized amine dehydrogenase and glucose dehydrogenase for asymmetric synthesis of amines by reductive amination with cofactor recycling. ChemCatChem.

[CR26] Liu L, Wang D-H, Chen F-F, Zhang Z-J, Chen Q, Xu J-H, Wang Z-L, Zheng G-W (2020). Development of an engineered thermostable amine dehydrogenase for the synthesis of structurally diverse chiral amines. Catal Sci Technol.

[CR27] Mattey AP, Ford GJ, Citoler J, Baldwin C, Marshall JR, Palmer RB, Thompson M, Turner NJ, Cosgrove SC, Flitsch SL (2021). Development of continuous flow systems to access secondary amines through previously incompatible biocatalytic cascades. Angew Chem-Int Ed Engl.

[CR29] Mayol O, David S, Darii E, Debard A, Mariage A, Pellouin V, Petit J-L, Salanoubat M, de Berardinis V, Zaparucha A, Vergne-Vaxelaire C (2016). Asymmetric reductive amination by a wild-type amine dehydrogenase from the thermophilic bacteria Petrotoga Mobilis. Catal Sci Technol.

[CR28] Mayol O, Bastard K, Beloti L, Frese A, Turkenburg JP, Petit J-L, Mariage A, Debard A, Pellouin V, Perret A, de Berardinis V, Zaparucha A, Grogan G, Vergne-Vaxelaire C (2019). A family of native amine dehydrogenases for the asymmetric reductive amination of ketones. Nat Catal.

[CR30] Meyer LE, Hobisch M, Kara S (2022). Process intensification in continuous flow biocatalysis by up and downstream processing strategies. Curr Opin Biotechnol.

[CR31] Ming H, Yuan B, Qu G, Sun Z (2022). Engineering the activity of amine dehydrogenase in the asymmetric reductive amination of hydroxyl ketones. Catal Sci Technol.

[CR32] Otvos SB, Kappe CO (2021). Continuous flow asymmetric synthesis of chiral active pharmaceutical ingredients and their advanced intermediates. Green Chem.

[CR33] Plutschack MB, Pieber B, Gilmore K, Seeberger PH (2017). The Hitchhiker’s guide to flow chemistry(ii). Chem Rev.

[CR34] Pushpanath A, Siirola E, Bornadel A, Woodlock D, Schell U (2017). Understanding and overcoming the limitations of bacillus badius and caldalkalibacillus thermarum amine dehydrogenases for biocatalytic reductive amination. ACS Catal.

[CR35] Ren H, Zhang Y, Su J, Lin P, Wang B, Fang B, Wang S (2017). Encapsulation of amine dehydrogenase in hybrid titania nanoparticles by polyethylenimine coating and templated biomineralization. J Biotechnol.

[CR36] Reus B, Damian M, Mutti FG (2024). Advances in cofactor immobilization for enhanced continuous-flow biocatalysis. J Flow Chem.

[CR37] Romero-Fernandez M, Paradisi F (2021). Biocatalytic access to betazole using a one-pot multienzymatic system in continuous flow. Green Chem.

[CR38] Sheldon RA, Brady D (2019). Broadening the scope of biocatalysis in sustainable organic synthesis. Chemsuschem.

[CR39] Sokač Cvetnić T, Šalić A, Benković M, Jurina T, Valinger D, Gajdoš Kljusurić J, Zelić B, Jurinjak Tušek A (2023) A systematic review of enzymatic kinetics in microreactors. Catalysts 13. 10.3390/catal13040708

[CR40] Tamborini L, Fernandes P, Paradisi F, Molinari F (2018). Flow bioreactors as complementary tools for biocatalytic process intensification. Trends Biotechnol.

[CR41] Thompson MP, Derrington SR, Heath RS, Porter JL, Mangas-Sanchez J, Devine PN, Truppo MD, Turner NJ (2019). A generic platform for the immobilisation of engineered biocatalysts. Tetrahedron.

[CR42] Thompson MP, Penafiel I, Cosgrove SC, Turner NJ (2019). Biocatalysis using immobilized enzymes in continuous flow for the synthesis of fine chemicals. Org Process Res Dev.

[CR43] Tong F, Qin Z, Wang H, Jiang Y, Li J, Ming H, Qu G, Xiao Y, Sun Z (2021) Biosynthesis of chiral amino alcohols via an engineered amine dehydrogenase in e. coli. front bioeng biotechnol 9:778584. 10.3389/fbioe.2021.778584 10.3389/fbioe.2021.778584 PMC8766677 35071200

[CR44] Tseliou V, Knaus T, Masman MF, Corrado ML, Mutti FG (2019). Generation of amine dehydrogenases with increased catalytic performance and substrate scope from epsilon-deaminating L-Lysine dehydrogenase. Nat Commun.

[CR45] Tseliou V, Schilder D, Masman MF, Knaus T, Mutti FG (2021). Generation of oxidoreductases with dual alcohol dehydrogenase and amine dehydrogenase activity. Chem -Eur J.

[CR46] Wan L, Kong G, Liu M, Jiang M, Cheng D, Chen F (2022). Flow chemistry in the multi-step synthesis of natural products. Green Synth Catal.

[CR47] Wang H, Qu G, Li J-K, Ma J-A, Guo J, Miao Y, Sun Z (2020). Data mining of amine dehydrogenases for the synthesis of enantiopure amino alcohols. Catal Sci Technol.

[CR48] Ye LJ, Toh HH, Yang Y, Adams JP, Snajdrova R, Li Z (2015). Engineering of amine dehydrogenase for asymmetric reductive amination of ketone by evolving rhodococcus phenylalanine dehydrogenase. ACS Catal.

[CR50] Zhang XJ, Shi PX, Deng HZ, Wang XX, Liu ZQ, Zheng YG (2018). Biosynthesis of chiral epichlorohydrin using an immobilized halohydrin dehalogenase in aqueous and non-aqueous phase. Bioresour Technol.

[CR49] Zhang W, Li S-F, Zhu J-Q, Cao H-X, Liu H-T, Shao Z-Q, Xu S-Y, Wang Y-J, Zheng Y-G (2023) Constructing a continuous-flow bioreactor with co-immobilized KmAKR and BmGDH for synthesizing tert-butyl 6-cyano-(3R,5R)-dihydroxyhexanoate. Biochem Eng J 197. 10.1016/j.bej.2023.108964

[CR51] Zor C, Reeve HA, Quinson J, Thompson LA, Lonsdale TH, Dillon F, Grobert N, Vincent KA (2017). H(2)-Driven biocatalytic hydrogenation in continuous flow using enzyme-modified carbon nanotube columns. Chem Commun.

