# Supplementary figures and images for: Co-immobilization of amine dehydrogenase and glucose dehydrogenase for the biosynthesis of (S)-2-aminobutan-1-ol in continuous flow
Source: Bioresour Bioprocess. 2024 Jul 18;11(1):70. doi: 10.1186/s40643-024-00786-0 (PMC11258105; doi:10.1186/s40643-024-00786-0)

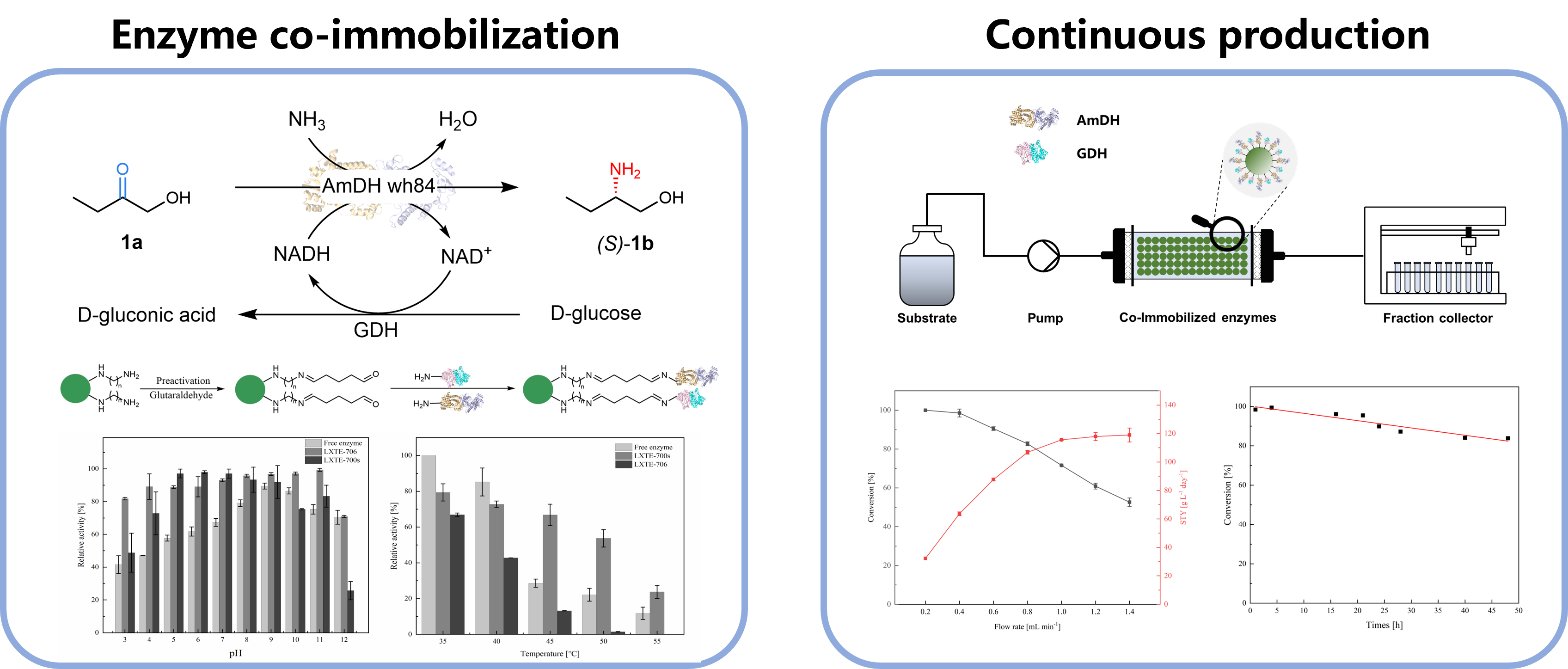

Supplement: Supplementary file 1 — Supplementary Material 1 [file 40643_2024_786_MOESM1_ESM.tif]
