# Supplementary material for: Co-immobilization of amine dehydrogenase and glucose dehydrogenase for the biosynthesis of (S)-2-aminobutan-1-ol in continuous flow
Source: Bioresour Bioprocess. 2024 Jul 18;11(1):70. doi: 10.1186/s40643-024-00786-0 (PMC11258105; doi:10.1186/s40643-024-00786-0)
Supplement: Supplementary file 2 — Supplementary Material 2 [file 40643_2024_786_MOESM2_ESM.docx]

**Co-immobilization of Amine Dehydrogenase and Glucose Dehydrogenase for the Biosynthesis of (*S*)-2-aminobutan-1-ol in Continuous Flow**

Pengcheng Xie^a, b, c^, Jin Lan^a, b, c^, Jingshuan Zhou^a, b, c^, Zhun Hu^d^, Jiandong Cui^a^, Ge Qu^b,c^, Bo Yuan^b,c,*^, Zhoutong Sun^b,c,*^

^a^ College of Biotechnology, Tianjin University of Science and Technology, Tianjin 300457, China

^b^ Tianjin Institute of Industrial Biotechnology, Chinese Academy of Sciences, Tianjin 300308, China

^c^ Key Laboratory of Engineering Biology for Low-Carbon Manufacturing, Chinese Academy of Sciences, 32 West 7th Avenue, Tianjin Airport Economic Area, Tianjin 300308, China

^d^ Institute of Industrial Catalysis, School of Chemical Engineering and Technology, Xi’an Jiaotong University, Xi’an Shaanxi 710049, China.

*Corresponding authors:

E-mail: yuanb@tib.cas.cn (B. Yuan); sunzht@tib.cas.cn (Z. Sun).

Number of pages: 16

Number of tables: 4

Number of figures: 8

Number of schemes: 1

**Table of Contents**

1. Sequence Information S3

2. Reactions in Batch S3

3. Continuous Reactions with a Fixed-bed Reactor S4

4. Supplementary Figures S5

5. Supplementary Tables S12

1. Sequence information

**Protein sequence of AmDH（wh84）:** MEIFKYMEHQDYEQLVICQDKASGLKAIIAIHDTTLGPALGGTRMWTYASEEEAIEDALRLARGMTYSNAAAGLNLGGGKTVIIGNPKTDKNDEMFRAFGRYIEGLNGRYFTACDVGTTEADMDLINLETDYVTGTSAGAGSSGNPSPVTAYGIYYGMKAAAKEAFGDDSLAGKTVAVQGVGNVAYALCEYLHEEGAKLIITDINEEAVQRAVDAFGATAVGINEIYSQEADIFAPCALGAIINDETIPQLKAKVIAGSALNQLKETRHGDLIHEMGIVYAPDYVINSGGVINCADELDGYNRERALKRVEGIYDVIGKIFAISKRDNIPTYVAADRMAEERIARVANTRSTFLQNEKSVLSRR

**Protein sequence of GDH:**

MYTDLKDKVVVITGGSTGLGRAMAVRFGQEEAKVVINYYNNEEEALDAKKEVEEAGGQAIIVQGDVTKEEDVVNLVQTAIKEFGTLDVMINNAGVENPVPSHELSLDNWNKVIDTNLTGAFLGSREAIKYFVENDIKGNVINMSSVHEMIPWPLFVHYAASKGGMKLMTETLALEYAPKGIRVNNIGPGAMNTPINAEKFADPVQRADVESMIPMGYIGKPEEVAAVAAFLASSQASYVTGITLFADGGMTKYPSFQAGRG

1. Reactions in batch

**Scheme S1**. General scheme for AmDH reactions in batch.

**Batch reactions of immobilized enzymes：**Reactions were performed under atmosphere. All enzymatic activity assays were performed in batch mode, where 200 mg immobilized enzyme was added to 1 ml Tris-HCl (including 1 M NH_4_Cl) buffer (pH 9.5) containing 10 mM **1a**, 1 mM NAD^+^ and 100 mM glucose. The mixture was then incubated with agitation at 30 ℃ and 220 rpm for 0.5 to 2 hours. The generated products were quantified using HPLC analysis.

**Batch reactions of crude enzymes：**Reactions were performed under atmosphere, where 200 µL crude enzyme was added to 1 ml Tris-HCl (including 1 M NH_4_Cl) buffer (pH 9.5) containing 10 mM 1a, 1 mM NAD^+^ and 100 mM glucose. The mixture was then incubated with agitation at 30 ℃ and 220 rpm for 0.5 to 2 hours. The generated products were quantified using HPLC analysis.

1. Continuous reactions with a fixed-bed reactor

Immobilized enzyme was filled in a fix-bed reactor (100 mm length, 10 mm diameter and 7.85 mL volume). A peristaltic pump was used to pump Tris-HCl (including 1 M NH_4_Cl) buffer containing 10 mM **1a**, 1 mM NAD^+^, and 100 mM glucose into the packed bed bioreactor. 2 column volumes (CV) of the solution mixture were allowed to flow through before the reactions reached steady state and conversions reached plateau. Subsequently, the samples were collected in the fraction collector and analyzed using HPLC.

1. Analysis of lysine distribution on the enzyme surface

The average protein concentration of prepared AmDH (wh84) and GDH crude enzyme is approximately 65 mg mL^-1^. The optimal volume ratio of immobilized AmDH (wh84) to GDH is 5:1. Using known protein molecular weights, it can be calculated that the amount of AmDH (wh84) per mL of crude enzyme mixture is approximately 1.2 µmol, and GDH is approximately 0.36 µmol. Analysis of lysine on the enzyme surface showed 34 lysine residues on AmDH (wh84) and 32 lysine residues on GDH **(Table S2)**. Thus, it can be calculated that each mL of AmDH (wh84) and GDH crude enzyme mixture contains approximately 52.32 µmol of total lysine residues. The functional group capacity of amino and epoxy resins is approximately 400 µmol g^-1^. Therefore, ideally, when the carrier-to-enzyme volume ratio is 1/8, it ensures all enzyme units are immobilized. However, the immobilization yield (IY) at this ratio is very low, possibly due to limited carrier binding capacity or weak binding of impurities in the protein crude extract, which are washed off during carrier cleaning. Hence, we set the carrier-to-enzyme ratio at 1/4 to ensure nearly 50% immobilization yield.

1. Supplementary figures

**
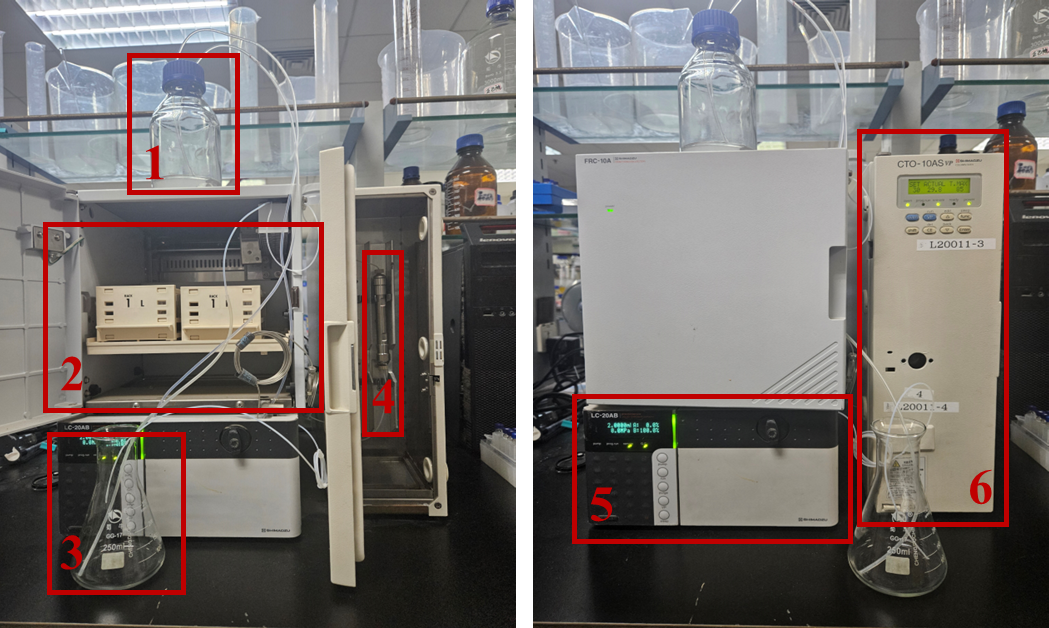
**

**Fig. S1.** Continuous flow reactor system components. 1, Reservoir (500 mL). 2, Fraction Collector. 3, Waste liquids bottle. 4, Fixed-bed reactor. 5, Pump. 6, Column Oven.

**
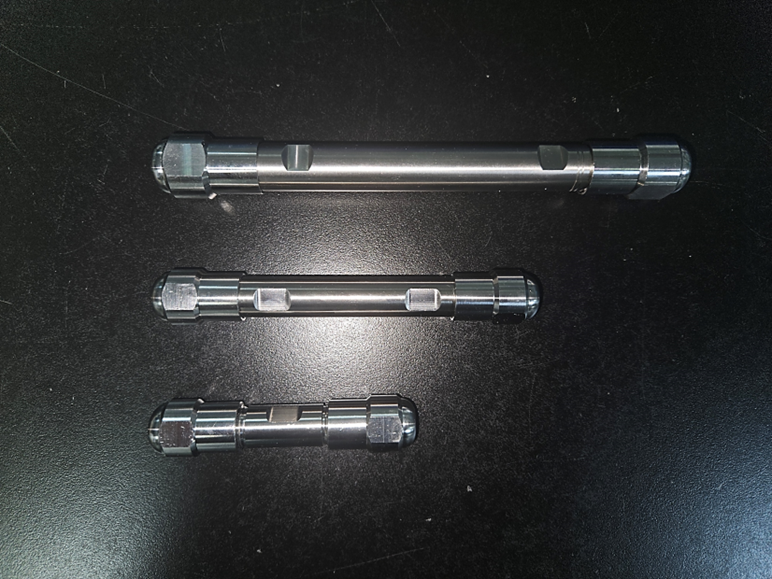
**

**Fig. S2.** Fixed-bed reactor. From bottom to top: Y1 (3.93 mL); Y2 (7.85 mL); Y3 (11.78 mL).


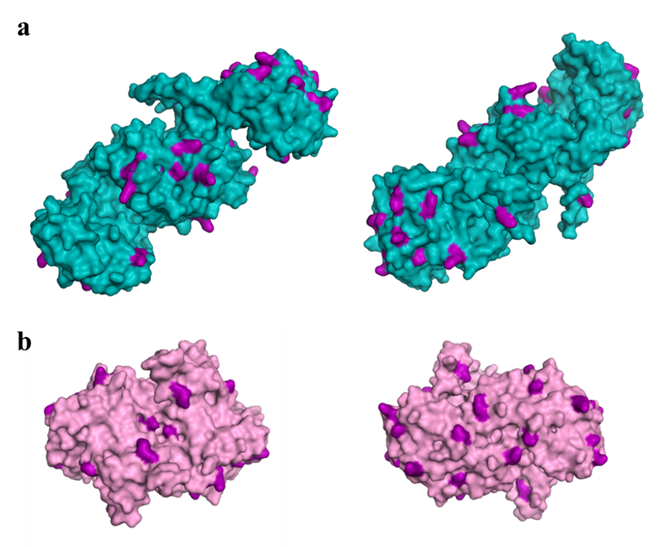


**Fig. S3.** Surface visualization of dimeric AmDH (PDB code: 3VPX) and GDH (PDB code: 3AUS). Residues in purple are Lysine residues. (a), AmDH surface Lysine distribution. (b), GDH surface Lysine distribution.

**
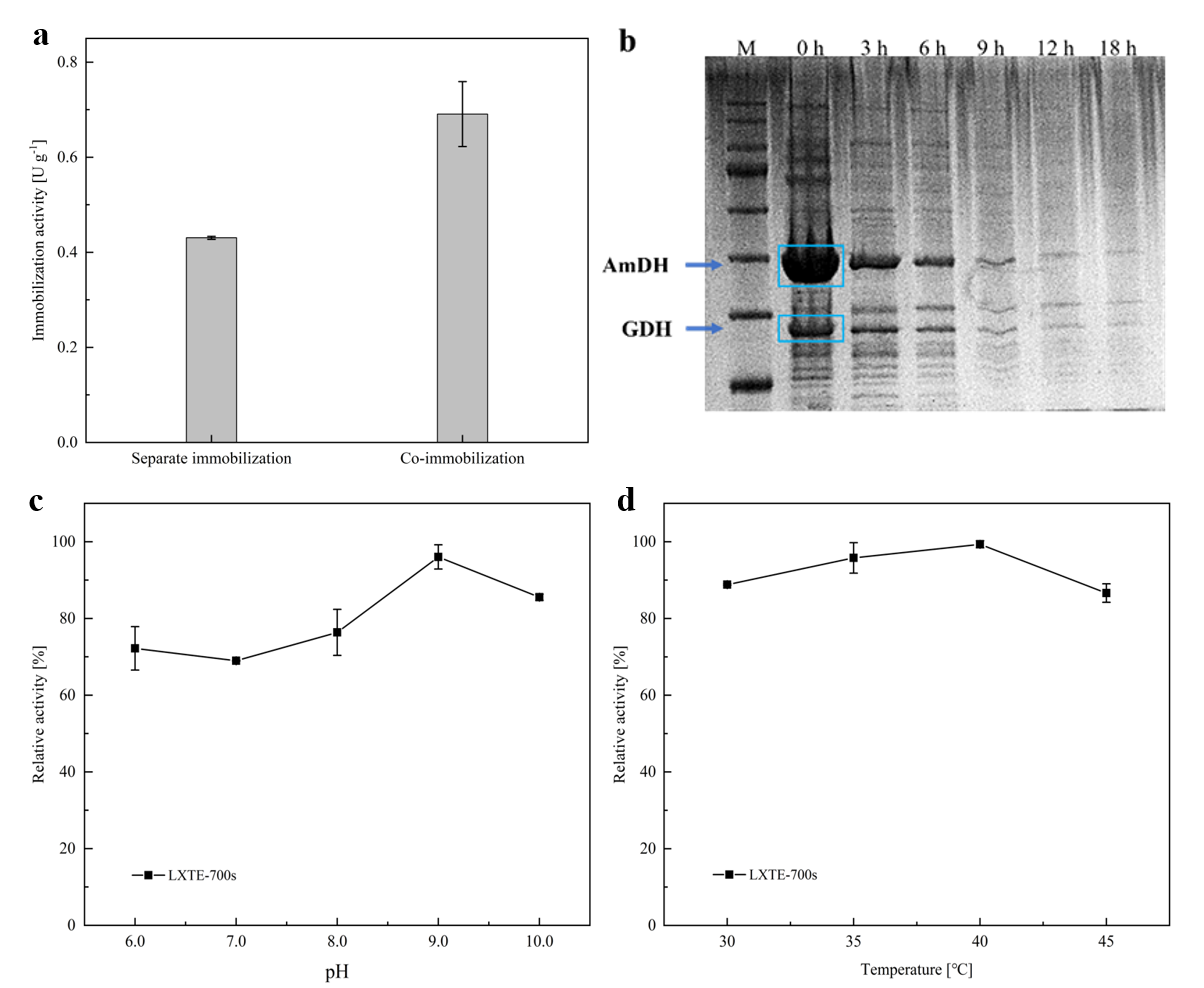
**

**Fig. S4.** Optimization of the batch reactions with co-immobilized enzymes. (a), The comparison of different immobilization strategies. Separate immobilization: AmDH and GDH were immobilized separately with LXTE-700s and then mixed in a 5:1 ratio as catalysts for the reaction; Co-immobilization: AmDH and GDH were mixed in a 5:1 ratio by volume and immobilized with LXTE-700s, which then served as a catalyst for the reaction. (b), The SDS-PAGE analysis of the crude enzymes before and after immobilization for 3-18 h. (c), Optimization of pH for the batch reactions using co-immobilized AmDH wh84 and GDH. Buffer pH was adjusted by KOH or HCl. Reaction conditions: NAD^+^ (1 mM), glucose (100 mM), Tris-HCl (50 mM, pH 6.0 - 10.0) buffer (including 1 M NH_4_Cl), immobilized enzymes (200 mg), 1a (10 mM), 30 °C, total volume: 1 mL, carrier: LXTE-700s.; (d), Optimization of reaction temperatures for the batch reactions using co-immobilized AmDH wh84 and GDH. Reaction conditions: NAD^+^ (1 mM), glucose (100 mM), Tris-HCl (50 mM, pH 9.5) buffer (including 1 M NH_4_Cl), immobilized enzymes (200 mg), 1a (10 mM), 30 - 50 °C, total volume: 1 mL, carrier: LXTE-700s.

**
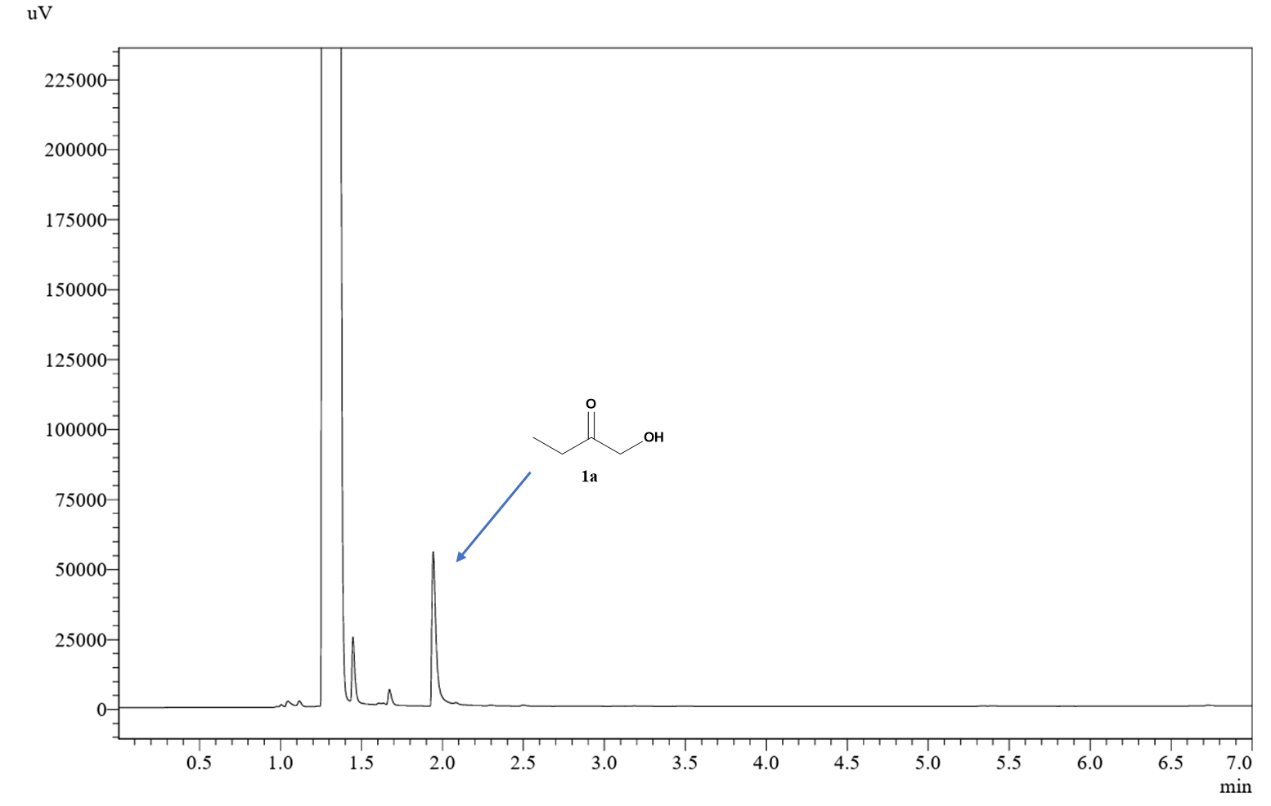
**

**Fig. S5.** GC standards for **1a**. (SH-Rtx-1, program: 50°C for 1.2 min; 30°C min^-1^ to 90°C for 2.0 min; 30°C min^-1^ to 310°C for 1.1 min, retention time [min] for **1a**: 1.873)


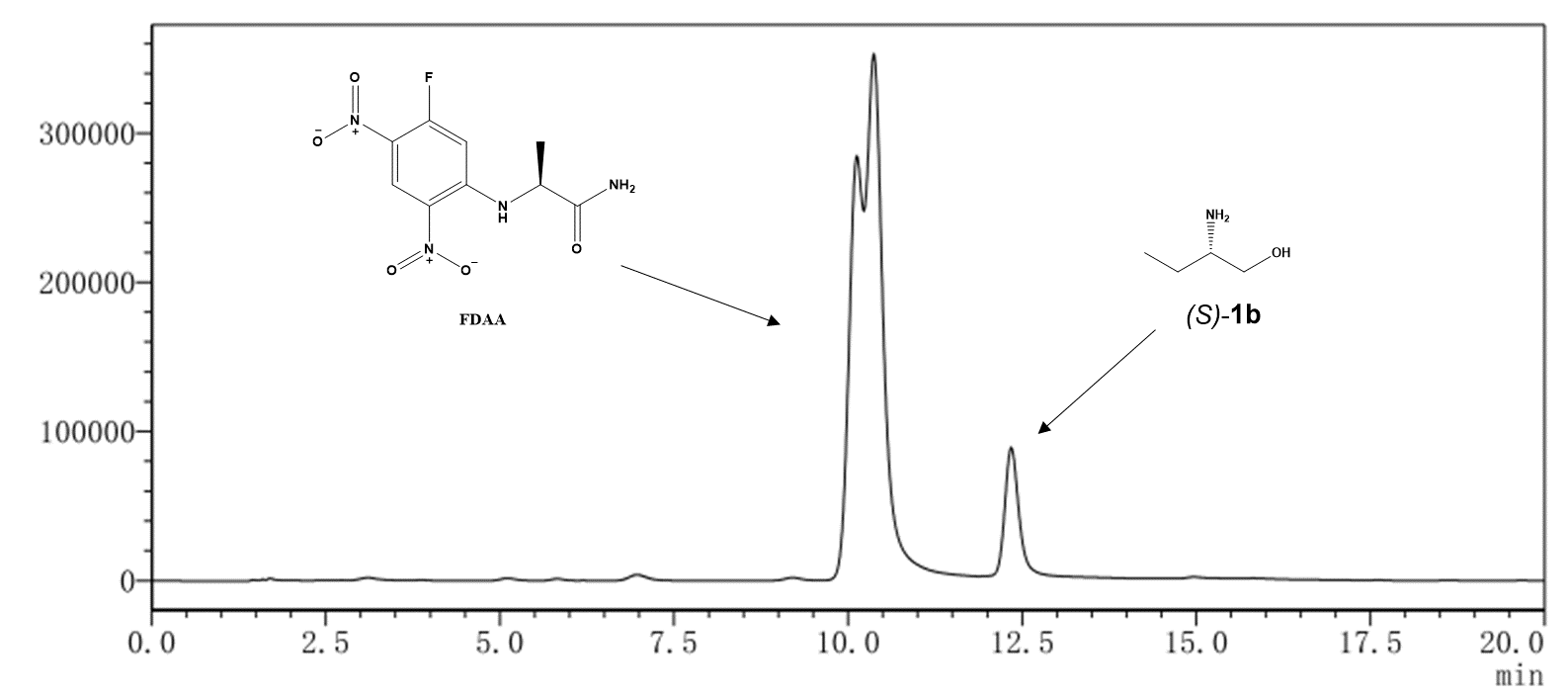


**Fig. S6.** HPLC spectra of (*S*)-**1b** synthesized from **1a** (10 mM) by co-immobilized AmDH (wh84) and GDH. (Zorbax SB-C18, mobile phase A: ultrapure water, mobile phase B: methanol, elution program: 60% A/40% B, hold at 40% B for 6 min, increase B to 60% within 9 min, hold for 3 min, then decrease B to 40% within 2 min, 340 nm, 25 °C, flow rate: 1.0 mL/min，retention time [min] for **1b**: 12.347 and FDAA:10.376


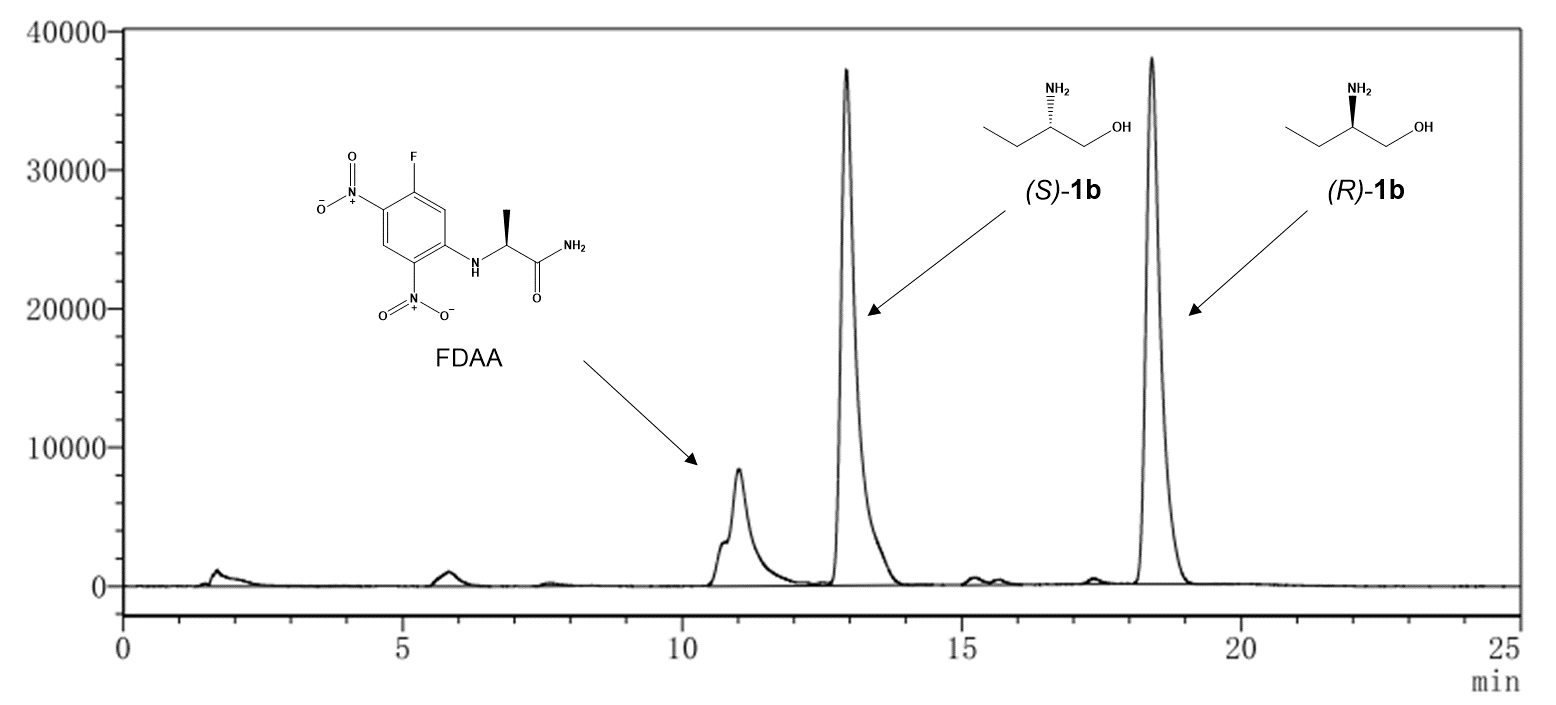


**Fig. S7**. HPLC spectra of mixed-spin standards for product **1b**. (Zorbax SB-C18, mobile phase A: ultrapure water, mobile phase B: methanol, elution program: 60% A/40% B, hold at 40% B for 6 min, increase B to 60% within 9 min, hold for 6 min, then decrease B to 40% within 4 min, 340 nm, 25 °C, flow rate: 1.0 mL/min，retention time [min] for (*S*)-**1b**: 12.939, (*R*)-**1b**: 18.403, FDAA:11.016

**
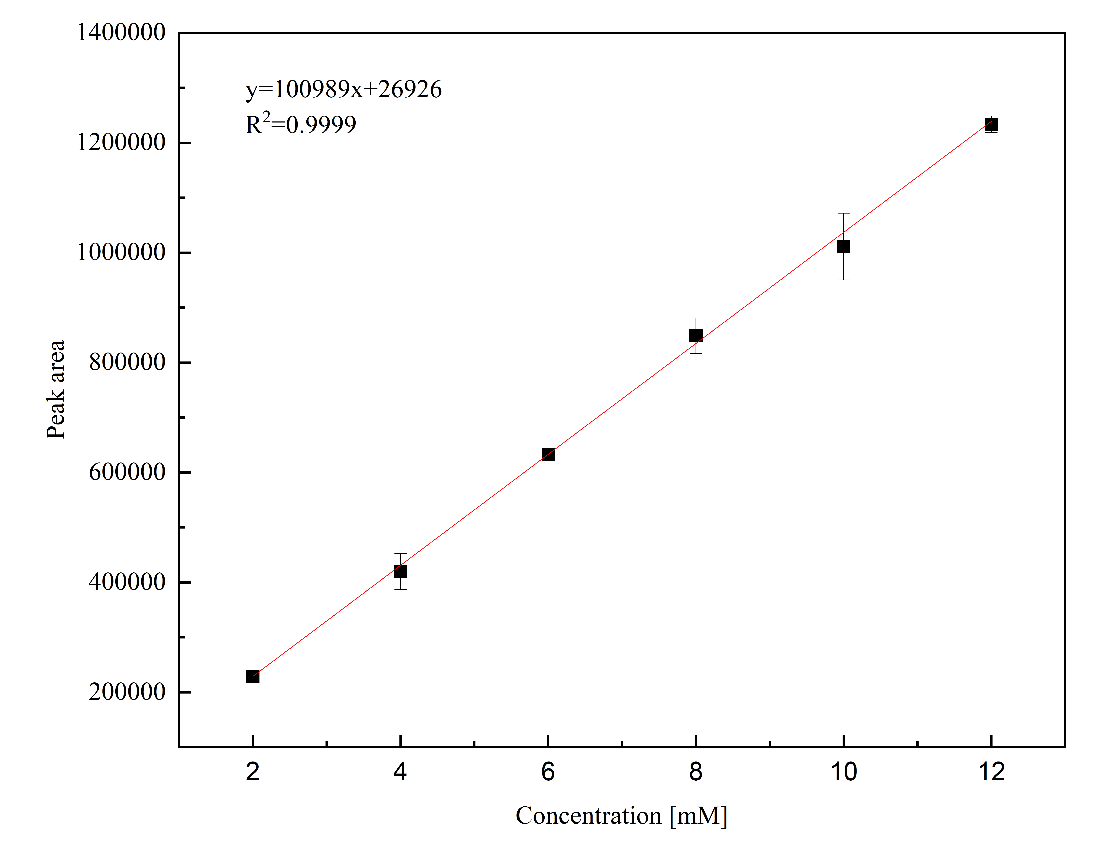
**

**Fig. S8.** Standard curve for the product (*S*)-**1b**.

1. Supplementary tables

**Table S1.** The comparisons of co-immobilized AmDHs and GDH on Magnetic nanoparticles.

| Carrier types | Models | Immobilization  activity (IA)^a^  [U g^-1^] | Activity  recovery (AR)^b^  [%] | Particle sizes  [µm] |
| --- | --- | --- | --- | --- |
| Magnetic  nanoparticles | SM-1000 | 0.014 | 0.6 | 1 |
|  | S-1000 | — | — | 1 |
|  | S-600 | 0.0039 | 0.2 | 0.4 |

a, IA was defined by Eq. (2); b, AR was defined by Eq. (1).

**Table S2**. Number of Lysines on the surface of AmDH and GDH

|  | AmDH | GDH |
| --- | --- | --- |
| Number of amino acids of the dimer | 728 | 522 |
| Number of surface Lysines | 34 | 32 |

**Table S3**. The average residence time for columns Y1-Y3 at various flow rates.

| Flow rates [mL min^-1^] | Residence Time [min] | | | |
| --- | --- | --- | --- | --- |
|  | Y1 (3.93 mL*) | Y2 (7.85 mL*) | Y3 (11.78mL*) | |
| 0.2 | 19.63 | 39.25 | | 58.88 |
| 0.4 | 9.81 | 19.63 | | 29.44 |
| 0.6 | 6.54 | 13.08 | | 19.63 |
| 0.8 | 4.91 | 9.81 | | 14.72 |
| 1 | 3.93 | 7.85 | | 11.78 |
| 1.2 | 3.27 | 6.54 | | 9.81 |
| 1.4 | 2.80 | 5.61 | | 8.41 |
| 1.6 | 2.45 | 4.91 | | 7.36 |

* Column volumes of Y1, Y2 and Y3.

**Table S4.** STY of different immobilized enzymes

| Immobilized enzyme | STY  [g L^-1^ d^-1^] | Conversion [%] | Reference |
| --- | --- | --- | --- |
| AmDH (wh84) & GDH | 124.5 | 48.2 | This article |
| AmDH (wh84) & GDH | 64.6 | 99.0 | This article |
| Cal-AmDH & JM-GDH 101 | 60 | 96 | (Pushpanath et al., 2017) |
| ChiAmDH & CbFDH | 300 | 68 | (Thompson et al., 2019) |
| ChiAmDH & TeSADH | 13 | 30 | (Thompson et al., 2019) |
| cFL1-AmDH & Cb-FDH | 433 | ~20 | (Franklin et al., 2021) |

**Reference**

Franklin RD, Whitley JA, Caparco AA, Bommarius BR, Champion JA, Bommarius AS (2021) Continuous production of a chiral amine in a packed bed reactor with co-immobilized amine dehydrogenase and formate dehydrogenase. Chem. Eng. J 407:1385-8947. <https://doi.org/10.1016/j.cej.2020.127065>

Pushpanath A, Siirola E, Bornadel A, Woodlock D, Schell U (2017) Understanding and Overcoming the Limitations of Bacillus badius and Caldalkalibacillus thermarum Amine Dehydrogenases for Biocatalytic Reductive Amination. ACS Catal 7:3204-3209. <https://doi.org/10.1021/acscatal.7b00516>

Thompson MP, Derrington SR, Heath RS, Porter JL, Mangas-Sanchez J, Devine PN, Truppo MD, Turner NJ (2019) A generic platform for the immobilisation of engineered biocatalysts. Tetrahedron 75:327-334. <https://doi.org/10.1016/j.tet.2018.12.004>
